# Identifying MSM-competent physicians in China: a national online cross-sectional survey among physicians who see male HIV/STI patients

**DOI:** 10.1186/s12913-018-3781-7

**Published:** 2018-12-13

**Authors:** Peipei Zhao, Bolin Cao, Cedric H. Bien-Gund, Weiming Tang, Jason J. Ong, Yi Ding, Weiying Chen, Joseph D. Tucker, Zhenzhou Luo

**Affiliations:** 1 Shenzhen Nanshan Center for Chronic Disease Control, Shenzhen, China; 2 School of Media and Communication, Shenzhen University, Nanshan Block Huaming Road, 7, Shenzhen, 518052 China; 3 University of North Carolina Project-China, No. 2 Lujing Road, Guangzhou, 510095 China; 4 School of Medicine, University of North Carolina at Chapel Hill, North, Carolina USA; 5 Faculty of Infectious and Tropical Diseases, London School of Hygiene and Tropical Medicine, London, UK; 6 Central Clinical School, Monash University, Melbourne, Australia

**Keywords:** Men who have sex with men, Clinical services, HIV care continuum, HIV/STI testing

## Abstract

**Background:**

Men who have sex with men (MSM) are at high risk of human immunodeficiency virus (HIV) infection and sexually transmitted infection (STI) in China. Inadequate clinical services and poor clinical competency among physicians are major barriers to improving the sexual health of MSM. This study aims to understand physician clinical competency in providing MSM health services in China.

**Methods:**

We conducted an online cross-sectional survey among Chinese physicians who have seen male patients for STI complaints in the past year. We obtained information on individual demographics, clinical practice, attitudes toward MSM, and interest in contributing to MSM clinical services. We defined an MSM-competent physician as one who asked male patients about sexual orientation, sexual practices, and recommended HIV/ STI testing during a clinic visit. We conducted multivariable logistic regression to identify factors associated with MSM competency.

**Results:**

In total, 501 physicians completed the survey. The most common subspecialties were dermatovenereology (33.1%), urology (30.1%), and general medicine (14.4%). Roughly half (*n* = 267, 53.3%) reported seeing MSM in the past 12 months. Among physicians who saw MSM in the past 12 months, 60.3% (*n* = 161) met criteria as MSM-competent physicians, and most (*n* = 234, 87.6%) MSM-competent physicians reported positive or neutral attitudes towards MSM. Over 60% of all physicians were willing to participate in activities for improving MSM services, such as training and being on a list of physicians willing to serve MSM. MSM-competent physicians showed no sociodemographic differences compared with non MSM-competent physicians. MSM-competent physicians were more willing to have their medical institution named on a public clinic list capable of serving MSM (aOR: 1.70, 95%CI: 1.01–2.86) and being on a public physician list capable of serving MSM (aOR: 1.77, 95%CI: 1.03–3.03).

**Conclusions:**

MSM-competent physicians included a broad range of individuals that practiced in diverse clinical settings. Most physicians were interested in improving and expanding MSM clinical services, despite having neutral attitudes toward same-sex behavior. Future interventions should focus on developing MSM clinical competency and expanding services that meet the needs of MSM.

## Background

Globally, men who have sex with men (MSM) are at elevated risk of HIV acquisition, other sexually transmitted infections (STIs), substance abuse, and mental health disorders [1–4]. In many low- and middle-income countries (LMICs), MSM face significant barriers to accessing health care [5, 6]. MSM in LMICs report stigma, discrimination, and ignorance of health services as major barriers to health care provision [7, 8]. In addition, MSM have reported negative interactions with physicians, such as providers who did not ask or disapproved of same-sex practices, did not provide sexual health counseling or HIV/STI testing services, or revealed their sexual orientation to others [9].

HIV prevalence among MSM in China has increased from 6.0% in 2010, to 8.0% in 2015 [10]. It was estimated that the lifetime HIV testing for MSM is 47%, and that 18% of them didn’t retrieve their results [11]. The infection risk of STIs was significantly higher in MSM compared to the general population [12]. The rapid spread of HIV and STIs among MSM has led to renewed efforts to improve health services for MSM [13]. However, Chinese policy towards MSM is ambiguous (no approval, no disapproval and no promotion) and same-sex marriage is illegal. In China, there is a lack of laws to protect MSM from discrimination and unfair treatment. Experienced and anticipated health discrimination is widely reported by MSM and is also a barrier for MSM to use health services (including HIV testing) in China [14, 15]. In one cross-sectional study of MSM in China, only 16.3% had disclosed their sexual orientation to any health care professional [16], lower than in high-income countries [17, 18]. Incompetent health care providers also delay the testing and services of HIV and STIs for MSM. Provider-initiated HIV testing services is an effective way to increase the uptake of HIV testing [19]. But in China, STI care providers offered HIV testing to only 28.4% of their patients [20]. In addition to providing HIV/STI testing services, physicians serving MSM are responsible for obtaining a thorough sexual history, discussing sexual practices, partners, and conducting an appropriate physical examination [15, 21]. But these services were not common practice among Chinese physicians [20]. In qualitative studies, MSM in China have indicated the importance of knowledgeable, confidential, gay-friendly physicians in accessing sexual health services [22]. Furthermore, physicians need the material resources necessary to provide appropriate care for MSM, such as condoms, lubricants, and sexual health information for patients [23].

MSM-competent health services are seen as a key component in HIV and STI prevention programs, which offers MSM comprehensive sexual health services including health promotion, counselling, peer support, prevention, adequate diagnostics and treatment. It provides health care that is sensitive to the special needs of MSM and encourages them to obtain health care services and disclosure sexual orientation to health providers [24]. Health providers, including physicians and nurses, need education and training to be able to deliver competent services. However, there is a lack of competency training among China’s health providers to provide more appropriate routine care and remove barriers for MSM to get appropriate services [20].

Physicians’ clinical competency to serve MSM has been overlooked in HIV and STI prevention programs. In order to inform the development of MSM-competent clinical care, we conducted a survey among Chinese physicians who have provided STI care to men. Given that it is difficult to evaluate the physicians’ competency in a comprehensive way, our study focused on their clinical behaviors. In this study, we adopted the definition of MSM-competent physicians from other original research as those who self-reported having asked their last male STI patient about sex with other men, anal sex, and recommended both HIV and syphilis testing [25]. We also assessed the physicians’ workplace information, their attitudes towards MSM and their willingness in future training to explore factors that correlate with competency.

## Methods

### Study design and participants

We conducted a cross-sectional, nationwide online survey of physicians who had seen male STI patients in China in the past twelve months. Physicians were recruited through the *Xingren* medical mobile phone application (app), where licensed physicians can register as online physicians on the app. The app allows registered physicians to communicate with patients, manage cases, and share medical knowledge and experiences with other physicians. Verified physicians in the app can get paid from their online encounters with patients by providing medical advice to patients. App-based counseling typically involves obtaining a clinical history, but without providing physical examination.

The survey link with an invitation message was sent to a subset of *Xingren* verified physicians who registered in dermatovenereology, urology, proctology, internal medicine, pediatrics, infectious disease, and general practice specialties (*n* = 120,126). Based on the profile of the registered physicians in the app, they were located all over mainland China. Physicians were included from both hospitals and primary care centers. In order to be eligible for the survey, physicians needed to live in China, have used the app within the last six months, and have seen at least one male STI patient offline in the past 12 months. The physicians registered in the Xingren app all had practice licenses in China. Male STI patients were defined as those who were diagnosed with at least one type of STI or had symptoms of STIs when they saw physicians. STIs include HIV, syphilis, chlamydia, gonorrhoea, human papillomavirus, herpes, trichomonas, and mycoplasma [26].

We pre-specified the recruitment sample as 500 participants. Based on previous research in China [27] we recruited a sample size of 500 physicians. This sample size was also limited by funding constraints as we provided an incentive for physicians to participate. We implemented a recruitment strategy in two steps. First, we sent the participation link to physicians whose unique *Xingren* identification number ended in 1, 2, or 3 (*n* = 6689). In order to meet the pre-specified sample size, we then sent the participation link to an additional 1400 physicians who were selected randomly from the rest of the eligible physicians. We used Randbetween function in Microsoft Excel to ensure that a random sample was invited. Physicians initiated the survey by clicking on the participation link. Participants were asked to sign an electronic consent form before they started the survey. We provided each participant with a small financial incentive for completion of the survey (~$4.50 USD).

### Survey

Eligible physicians completed a survey that included demographic and workplace information, clinical practice information, attitudes toward MSM, and interest in contributing to MSM clinic services.

#### Demographic and workplace information

We asked physicians their age, sex (male/female), highest educational degree (Associate/Bachelor/Master/PhD), and medical specialty. We also asked about the medical institution where physicians worked, including the level of care (primary, secondary or tertiary) and type of medical institution (public or private). The higher level of care means larger service coverage, more health functions and more medical practitioners. Higher level medical institution provided more comprehensive services. We asked for clinic information on medical equipment including the availability of proctoscopes or anoscopes (Yes/No), condoms and lubricants (Yes/No), and HIV/STI prevention pamphlets and educational materials (Yes/No).

#### Clinical practice information

Physicians were asked three items about their clinical practice during the last time they saw a male STI patient. These items included whether they asked about sex with other men, whether they asked about anal sex, and whether they recommended both HIV and syphilis testing. In this study, we defined MSM-competent physicians as those who self-reported having asked their last male STI patient about sex with other men, anal sex, and recommended both HIV and syphilis testing [25]. Although this is not a comprehensive benchmark for MSM clinical competence, it is consistent with previous literature on MSM health care [28–30]. In our analysis about MSM –competent physicians, we excluded those who have not seen MSM patients in the past 12 months.

#### Attitudes toward MSM

Physician attitudes toward MSM were measured by asking participants about their agreement with five statements. The 5-item scale was adapted from Herek’s Attitudes Toward Lesbians and Gay Men Scale [31], which has been validated in China [32]. Cronbach’s alpha value of the Chinese scale is 0.90 and confirmatory factor analysis demonstrates that it has good validity. The scale includes the following statements: “Male homosexuals are disgusting;” “Male homosexuality is a perversion;” “Homosexual behavior between two men is just plain wrong;” “Male homosexuality is merely a different kind of lifestyle that should not be condemned;” and “Homosexual should be segregated by society (residential segregation or occupational segregation).” The survey used a scale of 1 to 5 (1 = strongly disagree; 2 = disagree somewhat; 3 = neither agree nor disagree; 4 = agree somewhat; 5 = strongly agree).For each statement, 1 and 2 indicated positive attitudes; 3 indicate neutral attitudes; 4 and 5 indicate negative attitudes. Then items were summed to create a total score. Scores range from 5 (extremely positive attitude) to 25 (extremely negative attitude), with a value of 15 considered neutral. Those who scored less than 15 presented positive attitudes and more than 15 presented negative attitudes.

#### Interest in contributing to MSM clinical services

Physicians’ interest in contributing to quality improvement programs for MSM clinical services were also measured with three items. Physicians were asked whether they were interested in having their medical institution named on a public clinic list capable of serving MSM. In addition, physicians were asked about being included on a public physician list capable of serving MSM. They were also asked if they were interested in obtaining further training focused on clinical services for MSM.

##### Statistical analysis

IBM SPSS Statistics 19 was used for all analyses. Descriptive statistics were used to describe physicians’ socio-demographic information, institutional information, interests in contributing to the MSM clinical services and attitudes towards MSM. The primary outcome of the study is MSM-competent physicians, defined by physicians’ clinical practice with their last STI patients. Bivariate logistic regression was used to examine factors associated with being MSM-competent physicians. Demographic characteristics, workplace information, attitudes towards MSM and interested in contributing to MSM services are examined as independent variables. The results were reported as odds ratios (OR) with corresponding 95% confidence intervals (95%CI). Variables that were significant in the bivariate logistic regression were included in the multivariable analysis. In the multivariable logistic regression, we adjusted for demographic characteristics (age, sex, and education) and reported adjusted odds ratios (aOR). Statistical significance was determined at *P* < 0.05 in the model.

## Results

A total of 8089 physicians received the participation link, and 1556 physicians clicked the survey link and entered the survey, of which 699 provided informed consent. Among physicians who consented, 186 did not meet eligibility criteria, and another 12 surveys were invalidated due to insufficient information. A total of 501 physicians were included in the final analysis.

### Demographic characteristics and institutional information

On average, physicians in our survey were 37.6 ± 8.2 years old (Table 1). Three-quarters of them were male (*n* = 367, 75.0%), and over half of the physicians had obtained a master’s degree or higher (*n* = 276, 53.3%). One third of physicians reported specialization in dermatovenerology (*n* = 166, 33.1%), followed by urology (*n* = 151, 30.1%), and internal medicine (*n* = 51, 10.2%).

**Table 1:** Demographic characteristics of physicians who saw at least one male STI patient, 2017 (*N* = 501)

Characteristics	Total	
	*n*	%
Age (years)	Mean: 37.6 ± 8.2; Min: 23; Max: 76	
≤30	110	22.0%
31–40	244	48.7%
> 40	147	29.3%
Sex		
Male	376	75.0%
Female	125	25.0%
Education		
Associate’s degree^a^	36	7.2%
Bachelor’s degree	198	39.5%
Master’s degree	216	43.1%
PhD degree	51	10.2%
Specialty		
Dermatovenerology	166	33.1%
Urology	151	30.1%
General medicine^b^	72	14.4%
Proctology	41	8.2%
Others^c^	37	7.4%
Infectious disease	34	6.8%
Level of care		
Primary	34	6.8%
Secondary	145	28.9%
Tertiary	322	64.3%
Type of Medical institute		
Public	449	89.6%
Private	52	10.4%
Proctoscope or anoscope available		
Yes	403	80.4%
No	98	19.6%
Free condom and lubricants available		
Yes	260	51.9%
No	241	48.1%
STI prevention pamphlets or educational materials available		
Yes	377	75.2%
No	124	24.8%
Had seen MSM STI patients in the last 12 months		
Yes	267	53.3%
No	234	46.7%
Asked about sex with other men when seeing last patient		
Yes	378	75.4%
No	123	24.6%
Ask about anal sex when seeing last patient		
Yes	277	55.3%
No	224	44.7%
Recommended STI testing^d^ when seeing last patient		
Yes	455	90.8%
No	46	9.2%

^a^Associate’s degree is usually earned in two years or more and can be attained at community colleges, technical colleges, vocational schools, and some colleges;

^b^General medicine includes internal medicine, pediatrics and general practice;

^c^Others include Reproductive Medicine, Andrology, Emergence clinic, Hematology, Professional Health, AIDS Prevention Office;

^d^STI testing means both HIV testing and Syphilis testing

Almost two-thirds of the physicians worked in tertiary care hospitals (*n* = 322, 64.3%) and 89.6% (*n* = 449) worked in public medical institutions. Most physicians reported that proctoscopes or anoscopes were available in their medical facilities (*n* = 403, 80.4%), 75.2% (*n* = 377) reported STI prevention pamphlets or educational materials were available, and 51.9% (*n* = 260) reported free condom and lubricants were available on site.

### Physician clinical practice

In their last clinical encounter with a male STI patient, 75.4% (*n* = 378) of physicians reported asking about sex with other men. Over half (*n* = 277, 55.3%) asked about anal sex, and 90.8% (*n* = 455) recommended both HIV and syphilis testing.

### Physician attitudes to MSM

When presented with individual statements regarding MSM (Table 2), most physicians indicated they had neutral attitudes toward MSM. Nonetheless, the total scores of the scale showed most (*n* = 158, 59.2%) physicians surveyed had a positive attitude towards MSM. Nearly a third (*n* = 77, 29.2%) had neutral attitudes, and 11.6% (*n* = 33) had negative attitudes toward MSM.

**Table 2:** Physicians’ attitudes towards male homosexuals in China, 2017 (*N* = 267)

	Positive Attitudes	Neutral Attitudes	Negative Attitudes
Statements	*n* (%)	*n* (%)	*n* (%)
Overall	158(59.2%)	78(29.2%)	31(11.6%)
Male homosexuals are disgusting	60(22.5%)	180(67.4%)	37(10.1%)
Male homosexuality is a perversion	77(28.8%)	166(62.2%)	24(9.0%)
Homosexual behavior between two men is just plain wrong	73(27.3%)	155(58.1%)	39(14.6%)
Male homosexuality is merely a different kind of lifestyle that should not be condemned	113(42.3%)	124(46.5%)	30(11.2%)
Male homosexuals should be segregated by society (residential segregation; occupational segregation)	147(55.1%)	113(42.3%)	7(2.6%)

### Physician interest in contributing to improving medical services for MSM

When asked about interest in contributing to MSM clinical services, 59.5% (*n* = 298) reported they were interested in having their medical institution names on a public clinic list capable of serving MSM, 61.3% (*n* = 307) reported they were interested being on a public physician list capable of seeing MSM, and 68.1% (*n* = 341) of physicians were interested in further training focused on clinical services for MSM.

### Factors associated with being an MSM-competent physician

Among the 267 physicians who saw MSM STI patients, 60.3% (*n* = 161) met criteria as MSM-competent physicians. Among those who did not meet criteria, most (*n* = 98, 92.5%) failed to ask about anal sex with their patients. There were statistically significant differences between MSM-competent group with the non MSM-competent group in terms of age, sex, education, and specialty (Table 3). As for the level and type of the medical institution, there was no difference between MSM-competent and non-MSM-competent physicians. We did not observe any correlation between attitudes toward MSM and MSM-competency among physicians in our sample.

**Table 3:** Factors associated with being a MSM-competent physician in China, 2017 (*N* = 267)

	Overall^a^ (*n* = 267)	MSM-competent physicians (*n* = 161)	Non MSM-competent physicians (*n* = 106)	OR (95%CI)	aOR^b^ (95%CI)
	*n* (%)	*n* (%)	*n* (%)		
Age (years)					
≤30	55(20.6%)	27(16.8%)	28(26.4%)	Ref	
30–44	127(47.6%)	79(49.1%)	48(45.3%)	1.71(0.90–3.23)	
> 44	85(31.8%)	55(34.2%)	30(28.3%)	1.90(0.95–3.79)	
Sex					
Male	195(73.0%)	121(75.2%)	74(69.8%)	1.31(0.76–2.26)	
Female	72(27.0%)	40(24.8%)	32(30.2%)	Ref	
Education					
Associate’s degree^c^	15(5.6%)	10(6.2%)	5(4.7%)	2.00(0.56–7.10)	
Bachelor’s degree	98(36.7%)	62(38.5%)	36(34.0%)	1.72(0.78–3.79)	
Master’s degree	120(44.9%)	72(44.7%)	48(45.3%)	1.50(0.70–3.22)	
PhD degree	34(12.7%)	17(10.6%)	17(16.0%)	Ref	
Specialty					
Dermatovenerology	109(40.8%)	65(40.4%)	44(42.3%)	1.85(0.47–7.26)	
Urology	63(23.6%)	41(25.5%)	22(20.8%)	2.33(0.57–9.57)	
Proctology	27(10.1%)	19(11.8%)	8(7.5%)	2.97(0.63–14.03)	
General medicine^d^	38(14.2%)	16(9.9%)	22(20.8%)	0.91(0.21–3.93)	
Infectious Disease	21(7.9%)	16(9.9%)	5(4.7%)	4.00(0.77–20.92)	
Others^e^	9(3.4%)	4(2.5%)	5(4.7%)	Ref	
Level of care					
Primary	13(4.9%)	8(5.0%)	5(4.7%)	Ref	
Secondary	65(24.3%)	33(20.5%)	32(30.2%)	0.65(0.19–2.18)	
Tertiary	189(70.8%)	120(74.5%)	69(65.1%)	1.09(0.34–3.45)	
Type of Medical institute					
Public	238(89.1%)	145(90.1%)	93(87.7%)	1.27(0.58–2.76)	
Private	29(10.9%)	16(9.9%)	13(12.3%)	Ref	
Proctoscope or anoscope available					
Yes	219(82.0%)	138(85.7%)	81(76.4%)	1.85(0.99–3.47)	
No	48(18.0%)	23(14.3%)	25(23.6%)	Ref	
Free condom and lubricants available					
Yes	150(56.2%)	101(62.7%)	49(46.2%)	1.96(1.19–3.22)**	2.01(1.21–3.34)**
No	117(43.8%)	60(37.3%)	57(53.8%)	Ref	Ref
STI prevention pamphlets or educational materials available					
Yes	208(77.9%)	138(85.7%)	70(66.0%)	3.09(1.70–5.61)***	3.10(1.68–5.73)***
No	59(22.1%)	23(14.3%)	36(34.0%)	Ref	Ref
Interested in having medical institution name on the public clinic list capable of serving MSM					
Yes	174(65.2%)	113(70.2%)	61(57.5%)	1.74(1.04–2.90)*	1.70(1.01–2.86)*
No	93(34.8%)	48(29.8%)	45(42.5%)	Ref	Ref
Interested in being on a public physician list capable of serving MSM					
Yes	182(68.2%)	118(73.3%)	64(60.4%)	1.80(1.07–3.04)*	1.77(1.03–3.03)*
No	85(31.8%)	43(26.7%)	42(39.6%)	Ref	Ref
Interested in further training focused on clinical services for MSM					
Yes	194(72.7%)	123(76.4%)	71(67.0%)	1.60(0.93–2.75)	
No	73(27.3%)	38(23.6%)	35(33.0%)	Ref	
Physicians’ attitudes towards male homosexual					
Positive attitude	157(58.8%)	90(63.2%)	67(55.9%)	Ref	
Neutral attitude	77(28.8%)	50(25.5%)	27(31.3%)	1.38(0.78–2.43)	
Negative attitude	33(12.4%)	21(11.3%)	12(28.8%)	1.30(0.60–2.83)	

^a^Physicians who have not seen MSM patients in the past 12 months were excluded;

^b^aOR controlled for age, sex, and education;

^c^Associate’s degree is usually earned in two years or more and can be attained at community colleges, technical colleges, vocational schools, and some colleges;

^d^General medicine includes internal medicine, pediatrics and general practice;

^e^Others include Reproductive Medicine, Andrology, Emergence clinic, Hematology, Professional Health, AIDS Prevention Office;

* p < 0.05; ** *p* ≤ 0.01; *** *p* ≤ 0.001

Medical institutions where free condoms and lubricants were available were more likely to have MSM-competent physicians (aOR = 2.01, 95%CI: 1.21–3.34). This was true for medical institutions with STI pamphlets and educational material to have more MSM-competent physicians working there. Physicians who were more interested in contributing to activities to improve MSM clinical services were more likely to be MSM-competent. Activities included having their medical institution names on a public clinic list capable of serving MSM (aOR: 1.70, 95%CI: 1.01–2.86), and being on a public physician list capable of serving MSM (aOR: 1.77, 95%CI: 1.03–3.03).

## Discussion

This study explores clinical competency and attitudes toward MSM among an online sample of physicians in China. This study contributes to the limited literature on clinical services for MSM in China by examining clinical competency in serving MSM patients and attitudes among physicians who see male STI patients. We found that most physicians reported asking about sexual orientation, recommending STI testing, and asking about anal intercourse in their last clinical encounters. We also found that most physicians were willing to attend MSM-focused clinical training and openly identify as physicians willing to see MSM.

Over 60% of physicians who reported seeing MSM STI patients met basic criteria for MSM competency. Our results showed that the percentage of asking about same-sex sexual practices was higher than a previous study in China, which reported only 11% of the physicians asked their patients occasionally or most of the time [20]. The percentage of physicians asking about sexual practices is lower than that reported in high-income country studies focused on physicians who see patients with sexual health concerns [33, 34]. In our study, nearly all physicians reported recommending HIV/syphilis testing for their last STI patient. Although there are no comparable data for HIV testing among HIV/STI patients, research showed that a lower percentage of US physicians reported offering HIV testing. Among general practitioners in the US whose patients were general population, 41.7% of the physicians offered HIV testing [35]. Among HIV care providers whose patients were partly HIV infected, 60% routinely offered HIV testing to their patients [36]. Further studies should be conducted to investigate outcomes when physicians are seeing general patients and explore factors associated with patients accepting HIV testing offered by physicians in China.

MSM-competent physicians practiced in a wide range of clinical settings and medical subspecialties. Although some studies have found higher quality STI care in subspecialist clinics [37, 38], we did not observe significant differences in MSM competency between subspecialists and general practitioners. Additionally, MSM-competent physicians were associated with institutional factors (providing condom, lubricants, STI prevention pamphlets or educational materials), rather than individual factors. Recommendations of educational materials are recommended in guidelines for HIV prevention and LGBT patients, reinforcing the office-based education effect [39]. Guidelines for care of LGBT patients state that LGBT patients often find clues to determine what information they are comfortable to share with health care providers [40]. The educational and prevention material may have a potential effect on the interaction between physicians and STI patients. In addition, these materials may be the results of the institutional support for sexual health issues and risk population, which have a positive effect on physicians’ competency. Provision of educational and prevention material can play a useful role in an MSM competency improvement program.

Physician clinical competency and attitudes are both critical toward engaging MSM in care. In our study, most physicians reported positive attitudes towards MSM, although based on individual statement alone, most physicians reported neutral attitudes. Our finding contrasts with previous Chinese research from 1101 physicians showing negative physician attitudes toward MSM and widespread discrimination [41]. The more inclusive societal attitudes towards LGBT population showed from a recent national survey may influence health providers’ attitudes [42]. We found no correlation between the physicians’ MSM competency and attitudes towards MSM, indicating the disjunction between clinical practice and personal attitudes. However, it is the practical competency and non-judgmental attitude that work together to optimize MSM’s well-being. Physicians in China need both training to improve clinical practice and reduce stigma to better serve the MSM population.

We also found that most physicians were willing to receive further training to better serve MSM. Training and education initiatives to improve knowledge, attitudes, and clinical competencies among physicians for serving MSM patients is essential to improving the health of sexual minorities [43]. In LMICs, increased capacity from training in providing non-stigmatizing and non-discriminatory services to MSM patients can improve clinical outcomes [44, 45]. The high interest in our study suggests that subsequent MSM training may be feasible. Further research is needed to find the effect of training interventions or programs for physicians to improve their clinical practice and attitudes towards MSM.

The study has several limitations. First, the study was based on physician self-report, introducing the potential for social desirability and recall bias [46]. Physicians were asked about their last patients to reduce the likelihood of the recall bias, but responses related to competent clinical practice and willingness to contribute to MSM services might be over reported due to the social desirability bias. However, self-report surveys have been widely used in the studies with physicians [33, 34, 37, 47], allowing a reasonable comparison to the literature. Second, we used a narrow definition of MSM-competency that focuses on physicians’ clinical practice. Another limitation of the definition is the lack of patients’ voice and experience of the competency. Third, we did not assess whether the last clinical encounter was a new patient or an established patient, which may also influence physician behaviors. For established patient, physicians may have known the sexual history, so they did not need to ask these questions again in their last encounter. In this case, it would not necessarily indicate they are not competent. Forth, physicians were recruited from a medical app online, and there may be selection bias because all survey participants were app users. Fifth, this was a cross-sectional study, so no causal relationship can be established. Sixth, we didn’t obtain the information on how the STIs were confirmed by physicians. There was a potential for invalidity of STI diagnosis.

## Conclusions

This study expands the limited literature on MSM service providers in China, providing a current perspective of MSM-competent physicians. We observed a diverse sample of physicians from a range of subspecialties and medical institutions in China meeting criteria for competent clinical care for MSM. Most physicians were interested in the training and improving MSM clinical services. More research on physicians’ clinical competency is strongly recommended to provide a more accurate and comprehensive description of MSM-competency. Further research is needed to explore novel methods of engaging physicians in MSM care and developing interventions aimed at physicians in order to improve MSM clinical competency.

## Abbreviations

App: Mobile phone application
HIV: human immunodeficiency virus
LMICs: Low- and middle-income countries
MSM: Men who have sex with men
STI: Sexually transmitted infection
